# Construction and Immunological Evaluation of an Adenoviral Vector-Based Vaccine Candidate for Lassa Fever

**DOI:** 10.3390/v13030484

**Published:** 2021-03-15

**Authors:** Meirong Wang, Ruihua Li, Yaohui Li, Changming Yu, Xiangyang Chi, Shipo Wu, Shulin Liu, Junjie Xu, Wei Chen

**Affiliations:** Laboratory of Vaccine and Antibody Engineering, Beijing Institute of Biotechnology, Beijing 100071, China; lullabywangmeirong@outlook.com (M.W.); ilovebones@163.com (R.L.); 18612736076@163.com (Y.L.); yuchangming@126.com (C.Y.); goodnightcxy@163.com (X.C.); nkskywushipo@126.com (S.W.); lslsjy2203@163.com (S.L.)

**Keywords:** Lassa virus, Lassa fever, vaccine, adenovirus, glycoprotein

## Abstract

Lassa virus (LASV) is a rodent-borne arenavirus circulating in West African regions that causes Lassa fever (LF). LF is normally asymptomatic at the initial infection stage, but can progress to severe disease with multiorgan collapse and hemorrhagic fever. To date, the therapeutic choices are limited, and there is no approved vaccine for avoiding LASV infection. Adenoviral vector-based vaccines represent an effective countermeasure against LASV because of their safety and adequate immunogenicity, as demonstrated in use against other emerging viral infections. Here, we constructed and characterized a novel Ad5 (E1-, E3-) vectored vaccine containing the glycoprotein precursor (GPC) of LASV. Ad5-GPC_LASV_ elicited both humoral and cellular immune responses in BALB/c mice. Moreover, a bioluminescent imaging-based BALB/c mouse model infected with GPC-bearing and luciferase-expressing replication-incompetent LASV pseudovirus was utilized to evaluate the vaccine efficacy. The bioluminescence intensity of immunized mice was significantly lower than that of control mice after being inoculated with LASV pseudovirus. This study suggests that Ad5-GPC_LASV_ represents a potential vaccine candidate against LF.

## 1. Introduction

Lassa virus (LASV) is a rodent-borne arenavirus endemic in West African regions. LASV can cause Lassa fever (LF), which infects an estimated 100,000–300,000 people annually [1,2]. While most cases of LF are asymptomatic or result in mild flu-like illness, 20% of infections result in multiorgan failure and death [3,4]. Since no approved treatments or vaccines are available, LASV represents a severe public health problem that is prevalent in many districts. Therefore, to develop a safe and effective vaccine against LF is a pivotal medical need [1,4,5,6,7].

LASV is an enveloped virus with an ambisense genome comprising two sections of single-stranded RNA. The large (L) segment encodes for the viral RNA-dependent RNA polymerase (RdRp) protein (termed L or LP) and the Z matrix protein, where the small (S) segment encodes the nucleoprotein (NP) and the glycoprotein complex (GPC) [8]. The envelope glycoprotein complex (GPC) exists on the surface of LASV as trimeric spikes, and is cleaved by the protease SKI-1/S1P into receptor-binding subunit GP1 and the transmembrane fusion-mediating subunit GP2. The cleavage of GPC is essential for the formation of infectious virions [9]. GPC is the sole membrane-anchored surface protein of LASV and, hence, the main target in antibody-based therapeutics and vaccine design.

In severe cases of LF, the immune response is supposedly immunosuppressive [10,11,12,13]. LASV infects antigen-presenting cells (APCs) and interferes with the maturation and activation of cells, leading to a subdued T-cell response [13,14]. While humoral responses against LASV are weak in early infection [15,16], neutralizing monoclonal antibodies can be detected after recovery for six months to two years, and these antibodies were observed to be protective in animal models against LF [17,18]. Therefore, an ideal vaccine candidate would feature both cellular and humoral response. Several GPC-based recombinant viral-vectored and DNA vaccine candidates were shown to be protective against lethal LF challenge in preclinical animal testing. They provided protection associated with LASV GPC-specific T-cell and antibody responses [6]. While an inactivated LASV vaccine failed to protect nonhuman primates (NHPs) from infection, the lack of protection is considered to be associated with a limited ability of the inactivated vaccine to induce a robust cellular immune response [19].

The Ad5 (E1-, E3-) vector has the ability to infect antigen-presenting cells (APCs), further contributing to upregulation of costimulatory molecules and facilitation of the presentation of antigens [20]. Therefore, according to the need for a safe LF vaccine that elicits an effective cellular immune response, we developed a vaccine against LF using the Ad5 vector platform. Both the humoral response and the cell-mediated immunity elicited by the Ad5-based vaccine were studied. Moreover, the protection efficacies of the Ad5-based vaccine were evaluated in a bioluminescent imaging-based mouse model in response to infection with GPC-bearing and luciferase-expressing replication-incompetent LASV pseudovirus.

## 2. Materials and Methods

### 2.1. Phylogenetic and Conservation Analyses of LASV GPC Amino Acid Sequence

The amino acid sequences of six different LASV strains available in GeneBank were analyzed. The GenBank accession IDs are as follows: AAF86701.1 (LP), ADU56610.1 (Nig08-04), ADU56614.1 (Nig08-A18), ADY11068.1 (Josiah), CCA30314.1 (AV), and AMR44577.1 (Togo). Evolutionary analyses were conducted in MEGA X [21,22]. The evolutionary history was inferred using the maximum likelihood method and Jones–Taylor–Thornton (JTT) matrix-based model [23]. All positions with less than 95% site coverage were eliminated, i.e., fewer than 5% alignment gaps, missing data, and ambiguous bases allowed at any position (partial deletion option). There were a total of 490 positions in the final dataset.

### 2.2. Construction of Ad5-GPC_LASV_

Ad5-GPC_LASV_ was generated according to our previously described protocol with modifications [24,25]. The GPC gene sequence of the LASV Josiah strain was codon-optimized for increasing expression in human cell lines and synthesized (Sangon Biotech, Shanghai, China). The gene of LASV GPC, including a Kozak sequence, was cloned into the shuttle plasmid pDC316. The identity of the resulting recombinant plasmid was confirmed by sequencing and named pDC316-LASV GPC. The mixture of shuttle plasmid (pDC316-LASV GPC) and backbone plasmid (pBHGloxΔE1, 3Cre) was transformed into HEK293 cells to generate Ad5-GPC_LASV_. The recombinant Ad5 was amplified in 293F cells, purified by ion-exchange chromatography (GE Healthcare, Little Chalfont, United Kingdom), and concentrated by ultrafiltration with 100K Amicon^®^ Ultra 15 mL Centrifugal Filters (Merck Millipore, Darmstadt, Germany). Ad5-GPC_LASV_ titers were determined through a plaque-forming assay using a 2% methyl cellulose overlay.

### 2.3. Western Blotting

The expression of LASV GPC by Ad5 vectors was detected by Western blot. Briefly, 293T cells in a six-well cell culture plate were infected with Ad5-GPC_LASV_. The culture supernatant was discarded, and cells were washed twice with ice-cold phosphate-buffered saline (PBS) pH 7.4 and lysed by adding 200 µL of Pierce^®^ IP Lysis Buffer (Thermo Scientific, Waltham, MA, USA) containing Halt Protease and a Phosphatase Inhibitor Cocktail (Thermo Scientific, USA) to each well after 48 h. After centrifugation, the supernatant was collected, mixed with 6× Protein Loading Buffer (TransGen Biotech, Beijing, China), and 30 µL of each sample was separated on a 4–12% Bis-Tris protein gel (GenScript, Nanjing, China). The protein was transferred to a nitrocellulose membrane and probed with a primary antibody and then secondary antibody, i.e., anti-GPC (Lassa virus) antibody (Immune Technology, New York, NY, USA) and horseradish peroxidase (HRP)-conjugated goat anti-rabbit immunoglobulin G (IgG) antibody (Abcam, Cambridge, UK), respectively. The membranes were developed using SuperSignal™ West Pico PLUS Chemiluminescent Substrate (Thermo Scientific, USA), and imaged on an iBright Imaging System (Thermo Scientific, USA). β-Actin was detected on the same membrane stripped by Restore™ Western Blot Stripping Buffer (Thermo Scientific, USA) using HRP-conjugated anti-β-actin antibody (Abcam, Cambridge, UK).

### 2.4. Production of 37.7H

The 37.7H human antibody neutralizes LASV by stabilizing GPC in the prefusion conformation [26,27]. The variable region of 37.7H (PDB-5vk2) was combined with the human IgG1 constant region [26]. The genes of the light chain and heavy chain were codon-optimized, synthesized, and cloned into the pcDNA3.1 (+) vector. The plasmids of 37.7H were co-transfected with equal amounts into Expi293 cells (Thermo Fisher Scientific, USA). Lastly, 37.7H was purified using a HiTrap protein G HP affinity chromatography column (GE Health Care, Boston, MA, USA).

### 2.5. Flow Cytometry with 37.7H

Ad5-GPC_LASV_ infected and uninfected 293T cells were probed for prefusion LASV-GPC trimers with 37.7H by flow cytometry at 48 h post infection. Cells were detached with 4 mM ethylenediaminetetraacetic acid (EDTA) in PBS and stained with the LIVE/DEAD^TM^ Fixable Near-IR Dead Cell Stain Kit (Invitrogen, Carlsbad, CA, USA) and fluorescein isothiocyanate (FITC)-conjugated 37.7H. Flow cytometry was performed using a BD FACSCanto, and the data were analyzed with FlowJo software (BD, New York, NY, USA) using the following gating strategy: Single cells > size and granularity > live cells > 37.7H-FITC^+^.

### 2.6. Cell-to-Cell Fusion Assay

Forty hours after infection with Ad5-GPC_LASV_, 293T cells were treated with PBS (pH 4) and incubated at 37 °C in 5% CO_2_ for 30 min to enable glycoprotein triggering [28]. PBS was replaced with complete Dulbecco’s modified Eagle medium (DMEM), and cells were incubated for an additional 4 h to allow membrane rearrangement and syncytium formation. Pictures of the fusion were taken using Cytation 5 (BioTek Instruments, Inc., Winooski, VT, USA).

### 2.7. Recombinant LASV rGPe Protein Preparation

The recombinant linked ectodomain (residues 1–424) of LASV GPC (rGPe) [27,29] was generated by mutating the putative S1P cleavage site 256-RRLL-259 to 256--LLLL-259. rGPe was fused to an enterokinase cleavage site followed by a dual strep II tag and cloned into the pcDNA3.1 vector for transient expression in Expi293 cells (Thermo Fisher Scientific). The resulting protein was purified via StrepTrap HP affinity columns. Purified protein was concentrated using 10K Amicon^®^ Ultra 15 mL Centrifugal Filters (Merck Millipore, Darmstadt, Germany), and the buffer was replaced with PBS. The concentration of rGPe was determined using a Pierce™ bicinchoninic acid (BCA) Protein Assay Kit (Thermo Fisher Scientific, USA) and characterized using nonreduced SDS-PAGE.

### 2.8. Animal Experiments

The experiments involving animals were approved by and carried out in accordance with the guidelines of the Institutional Experimental Animal Welfare and Ethics Committee (approval number: IACUC of ZSBD-2018-A019-3R, date of approval is 4 March 2018). Specific pathogen-free female BALB/c mice aged 6–8 weeks were obtained from Beijing Vital River Laboratory Animal Technologies Co., Ltd. (Beijing, China) and were housed and bred in an animal facility with controlled temperature, humidity, and light cycles (20 ± 2 °C; 50 ± 10%; light, 7:00–19:00; dark, 19:00–7:00) at Sino Animal science and Technology Development Limited Company, Beijing. Mice were sacrificed at the indicated time by CO_2_ inhalation. All efforts were made to minimize suffering.

### 2.9. Vaccination of Mice

To evaluate the humoral response, BALB/c mice (*n* = 10 per group) were immunized intramuscularly with a dose of 10^6^/10^7^/10^8^ plaque-forming units (PFU) of Ad5-GPC_LASV_ once on day 0, or twice, on days 0 and 28. Sera were collected for LASV GPC-specific ELISA and LASV neutralizing antibody titration at 42 days post first vaccination.

For identification of CD8^+^ T-Cell epitopes, BALB/c mice (*n* = 3 per group) were immunized intramuscularly with a dose of 10^7^ PFU of Ad5-GPC_LASV_ once on day 0. The mice were sacrificed at 14 days postvaccination and their spleens were excised for enzyme-linked immunospot (ELISPOT) assay and intracellular cytokine staining.

For CD8^+^ T cell-mediated immune response evaluation, BALB/c mice (*n* = 10 per group) were immunized intramuscularly with a dose of 10^6^/10^7^/10^8^ plaque-forming units (PFU) of Ad5-GPC_LASV_ once on day 0, or twice, on days 0 and 28.Vaccinated mice were euthanized for a cluster of differentiation 8 (CD8)^+^ T-cell response analysis on days 14 and 42. The splenocytes were extracted into complete Gibco™ Roswell Park Memorial Institute (RPMI) 1640 Medium (GlutaMAX™ Supplement) (Thermo Scientific, USA) for intracellular cytokine staining.

### 2.10. ELISA

For LASV GPC-specific IgG assays, 96-well high-binding microplates (Corning, NY, USA) were coated with 1 µg/mL LASV rGPe protein in carbonate–bicarbonate buffer (pH 9.6) and incubated overnight at 4 °C. Then, the plates were blocked for 1 h at 37 °C in PBS containing 2% BSA and washed with PBST (PBS +0.1% Tween-20). Mice sera serially diluted in dilution buffer (PBS with 0.2% BSA) were added to the plates and incubated for 1 h at room temperature (RT). HRP-conjugated goat anti-mouse IgG (Abcam, UK) was diluted 10,000 times and added to the plates; then, the plates were incubated for 1 h and washed with PBST. The assay was developed for 10 min at RT in the dark using 100 µL of 3,3′,5,5′-tetramethylbenzidine (TMB) substrate solution (Solarbio, Beijing, China) and the reaction was halted by 50 µL stop solution (Solarbio, China), followed by measurement of emission at 450 nm (SPECTRA MAX 190, Molecular Device, San Jose, CA, USA). The endpoint titer was defined as the highest reciprocal serum dilution that yielded an absorbance ≥2.1-fold that of negative control serum values.

### 2.11. Prediction of H-2^d^-Specific CTL Epitopes in LASV GPC

H-2^d^-restricted epitopes of LASV GPC were predicted using IEDB (SMM), NetCTLpan, NetMHC, PREDEP, and ProPred-I (Table 1). Considering the scores and frequency of peptides in each prediction program, the most likely peptides were synthesized by Sangon Biotech Co., Ltd. (Shanghai, China) and stored at −80 °C until required.

### 2.12. ELISPOT Assay

Peptide-specific T cells were counted using a BD™ ELISPOT Mouse interferon (IFN)-γ Kit. ELISPOT plates were added along with 100 µL of diluted anti-mouse IFN-γ antibody (5 µg/mL) solution to each well and stored at 4 °C overnight. The plates were washed once and blocked for 2 h at room temperature with complete RPMI 1640 medium. Then, 100 µL of a solution with different peptides (20 µg/mL) and 100 µL of a splenocyte suspension (2 × 10^5^ cells) were added to ELISPOT plate microwells. The positive control concanavalin A (Con A; Sigma, Santa Clara, CA, USA) and a “no peptide” negative control were included in all assays. The plates were incubated for 24 h at 37 °C in a 5% CO_2_ humidified incubator. Following incubation, the wells were washed twice with deionized (DI) water and three times with washing buffer (PBS containing 0.05% Tween-20). Biotinylated anti-mouse IFN-γ was added to each well at a concentration of 2 µg/mL, and the plates were incubated for 2 h at RT. Following three washes, streptavidin–horseradish peroxidase was added to each well, and the plates were incubated for 1 h at RT. Following another four washes with PBST and three washes with PBS, spot development was monitored using a BD^TM^ ELISPOT AEC substrate set from 5 to 60 min. The substrate reactions were stopped by washing with DI water, followed by air-drying and analysis with an AT-Spot 3200 (Antai Yongxin Medical Technology, Beijing, China).

### 2.13. Intracellular Cytokine Staining and Flow Cytometry Analyses

The analyses were performed as previously described with modifications [30]. Briefly, murine splenocytes were stimulated for 6 h with peptide GL-9 dissolved in DMSO (2.5 µg/mL) or with an equal volume of DMSO as a negative control, and then incubated with BD GolgiStop^TM^ for 4 h. Following washing with PBS, cells were stained with the LIVE/DEAD^TM^ Fixable Near-IR Dead Cell Stain Kit (Invitrogen, USA). The cells were washed with PBS, blocked with Mouse BD Fc Block^TM^, and then incubated with anti-mouse antibodies (Biolegend, San Diego, CA, USA) including PerCP/Cy5.5 anti-mouse CD3 (clone 17A2), FITC anti-mouse CD8a (clone 5H10-1), and Brilliant Violet 421^TM^ anti-mouse CD107a (clone 1D4B), diluted in Cell Staining Buffer (Biolegend). Following simultaneous fixation and permeabilization with Cytofix/Cytoperm^TM^ Fixation and Permeabilization Solution (BD), cells were incubated with phycoerythrin (PE) anti-mouse IFN-γ (clone XMG1.2), APC anti-mouse TNF-α (clone MP6-XT22), and Brilliant Violet 605TM anti-mouse interleukin (IL)-2 (clone JES6-5H4) diluted in 1× Perm/Wash^TM^ Buffer (BD). The anti-rat/hamster Igκ/Negative Control Compensation Particle Set (BD) was used for fluorescence compensation. Flow cytometry was performed using a BD FACSCanto and analyzed by BD FACSDiva software.

### 2.14. LASV Pseudovirus and Neutralization Assay

LASV pseudovirus bearing the full-length GPC was generated in an Env-defective, luciferase-expressing replication-incompetent human immunodeficiency virus (HIV)-1 backbone. Briefly, pDC316-LASV-GPC and pNL4-3.Luc-R-E were co-transfected into 293T cells using transfection reagents Lipofectamine 3000 (Invitrogen, L3000015) or TurboFect (Thermo Scientific, R0531). The supernatants containing pseudovirus were harvested and supplemented with fresh medium at 24, 48, and 72 h. The LASV pseudovirus was filtered, aliquoted, and stored at −80 °C. For in vivo infection, the LASV pseudovirus was centrifuged at 50,000× *g* for 2 h at 4 °C and resuspended in PBS. Serial dilutions of heat-inactivated sera and titrated pseudovirus were co-incubated for 60 min at 37 °C and added along with the 293T cell suspension to 96-well microplates. Luciferase activity was measured using the Luciferase Assay System (Promega, Madison, WI, USA). Half maximal inhibitory concentration (IC_50_) neutralization titers were calculated for each individual mouse serum sample using the Reed–Muench method.

### 2.15. Efficacy Evaluation of Ad5-GPC_LASV_ against LASV Pseudovirus Infection and Bioluminescence Imaging (BLI) 

BALB/c mice (*n* = 3 per group) were immunized with a single intramuscular dose of 10^7^ PFU of Ad5-GPC_LASV_. After 4 weeks, mice were challenged with an intraperitoneal (i.p.) injection of 10^5^ median tissue culture infective dose (TCID_50_) LASV pseudovirus, and the photo flux was detected 1 day later. A bioluminescence image was acquired and analyzed with the IVIS Spectrum In Vivo Imaging System (PerkinElmer Inc., Waltham, MA, USA). Next, d-Luciferin Firefly (PerkinElmer), dissolved in PBS at a concentration of 15 mg/mL, was i.p. injected into each animal at a working dose of 150 mg/kg. Mice were placed into a Plexiglas anesthesia box (2.5–3.5% isoflurane) for 10 min before transferring them to the imaging chamber for in vivo imaging. Luminescence was measured over 5 min. The relative intensities of emitted light were presented as average radiance in p/s/cm^2^/sr.

## 3. Results

### 3.1. LASV GPC Amino Acid Sequences Are Highly Conserved among Six Lineages

**Figure 1:**
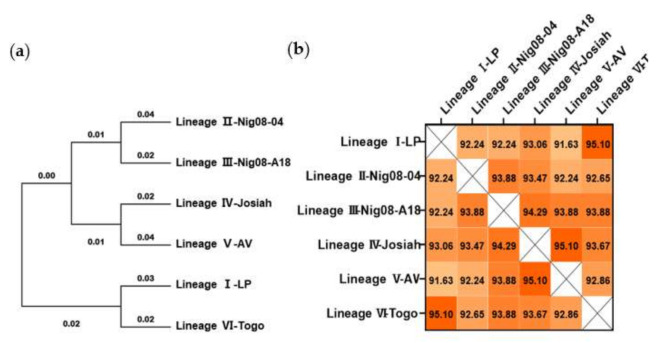
Phylogenetic and conservation analyses of Lassa virus (LASV) glycoprotein precursor (GPC) amino acid sequence. (**a**) Phylogenetic trees were inferred using the maximum likelihood method and Jones–Taylor–Thornton (JTT) matrix-based model. (**b**) Sequences were aligned by MUSCLE analysis, and the matrix for pairwise score similarity was converted into a heatmap. The GenBank accession IDs used for alignments are as follows: AAF86701.1 (LP), ADU56610.1 (Nig08-04), ADU56614.1 (Nig08-A18), ADY11068.1 (Josiah), CCA30314.1 (AV), and AMR44577.1 (Togo).

Bioinformatic analysis was conducted to identify the changes and differences in the amino acid sequence of GPC among six LASV strains from different lineages [31]. Phylogenetic analysis revealed a tree with very close branches (Figure 1a). In this tree, the LP strain (lineage I) and Togo strain (lineage VI) share a more common ancestor. The Nig08-04 strain (lineage II) was placed in a sister relationship with Nig01-A18 (lineage III). The prototype strain is Josiah (lineage IV), which clustered with strain AV (lineage V). This observation was recapitulated when comparing the amino acid sequence similarity of the six proteins (Figure 1b). The LP strain (lineage I) and Togo strain (lineage VI) showed the highest similarity at 95.10%, while the LP strain (lineage I) and AV strain (lineage V) showed the lowest similarity at 91.63%. The similarity between the Josiah strain (lineage IV) and all other strains is higher than 93%. Based on these results, the LASV GPC of the Josiah strain (lineage IV) was selected as the target for constructing vaccine candidates.

### 3.2. Construction and Characterization of Ad5-GPC_LASV_

**Figure 2:**
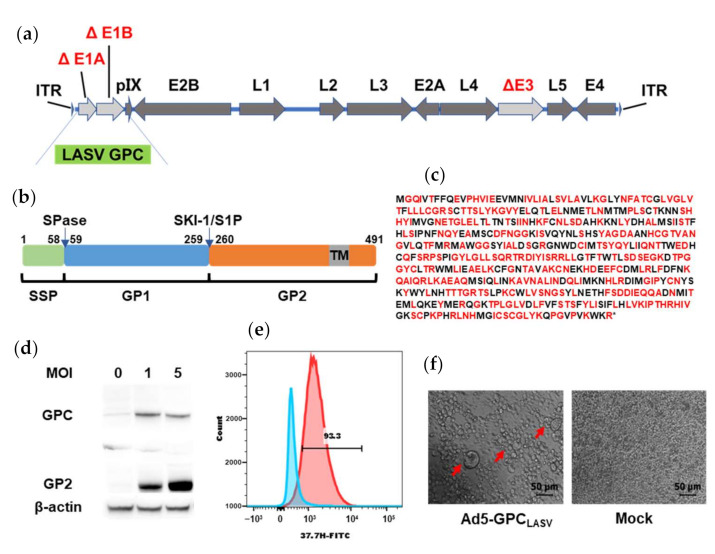
Generation and characterization of Ad5-GPC_LASV_. (**a**) Schematic of the adenovirus genome with mutations in the E1 and E3 regions. (**b**) Schematic presentation of LASV GPC. (**c**) Amino acid sequence of LASV GPC. Amino acid residues encoded by altered synonymous codons in the LASV GPC gene are indicated in red. (**d**) Expression of the LASV GPC in Ad5-GPC_LASV_-infected 293T cell lysates by Western blot analysis. (**e**) Ad5-GPC_LASV_-infected and uninfected 293T cells were probed for prefusion LASV GPC expression with 37.7H and analyzed by flow cytometry 48 h post infection. (**f**) Syncytium formation caused by LASV GPC expressed in Ad5-GPC_LASV_-infected cells after low-pH treatment.

The codon-optimized LASV GPC was inserted into the Ad5 (E1-, E3-) vector-based platform (Figure 2a–c). The expression of LASV GPC protein in 293T cells infected with Ad5-GPC_LASV_ at a multiplicity of infection (MOI) of 1 or 5 was confirmed via Western blot (Figure 2d). The most effective neutralizing antibodies were those directed against quaternary assemblies on the prefusion GPC. Hence, we evaluated the expression of prefusion LASV-GPC trimers using flow cytometry and a cell-to-cell fusion assay. Human monoclonal antibody (mAb) 37.7H can neutralize LASV by stabilizing GPC in the prefusion conformation [17,18,26,27]. Immunostaining with FITC-conjugated 37.7H was used for detection of LASV GPC on the cellular surface of 293T cells infected with Ad5-GPC_LASV_ (Figure 2e). Furthermore, LASV GPC expressed in Ad5-GPC_LASV_-infected cells induced extensive fusion and large syncytia following low-pH treatment (Figure 2f).

### 3.3. Quantification and Characterization of LASV GPC-Specific Antibodies

**Figure 3:**
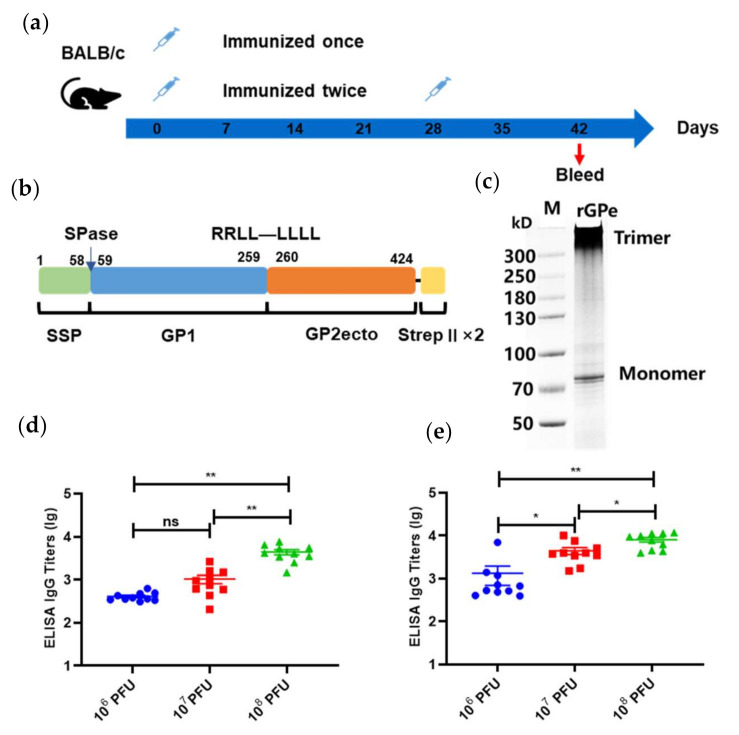
Humoral immune responses after vaccination with Ad5-GPC_LASV_. (**a**) BALB/c mice (*n* = 10 per group) were immunized intramuscularly with doses of 10^6^/10^7^/10^8^ plaque-forming units (PFU) of Ad5-GPC_LASV_ once on day 0, or twice, on days 0 and 28. Sera were collected on day 42 post first vaccination. (**b**) Schematic of linked recombinant GPC lacking the transmembrane and intracytoplasmic domains (rGPe). (**c**) LASV rGPe was expressed in the Expi293 suspension cells and purified by affinity chromatography. Purified proteins were assessed on nonreduced SDS-PAGE gel. Humoral immune responses were assessed via LASV GPC-specific ELISA from the sera of mice vaccinated once (**d**) and twice (**e**). Data are presented as mean ± SEM, and statistical significance was determined by one way ANOVA and Turkey’s multiple comparison test (* *p* < 0.05; ** *p* < 0.01).

According to our experimental design (Figure 3a), mice were immunized once on day 0, or twice, on days 0 and 28, with either 1 × 10^6^/10^7^/10^8^ PFU of the Ad5-GPC_LASV_ (*n* = 10). To determine the antibody levels after vaccination, sera collected on day 42 post first immunization were evaluated using enzyme-linked immunosorbent assay (ELISA) using a linked Expi293 cell-produced recombinant GPC lacking the transmembrane and intracytoplasmic domains (rGPe) [27,29] as the capture antigen (Figure 3b,c). The ELISA titers of sera from mice vaccinated once or twice with LASV GPC were 400–4000 and 1000–8000, respectively (Figure 3d,e). Vaccination either once or twice induced specific anti-LASV GPC-binding antibodies in a dose-dependent manner. The antibody titer of mice vaccinated twice was generally 0.5 to 1 log higher than the mice vaccinated once receiving the same dose of vaccine.

### 3.4. Prediction and Identification of H-2^d^-Restricted CD8^+^ T-Cell Epitopes in LASV GPC

The prediction of class I major histocompatibility complex (MHC)-restricted epitopes in LASV GPC sequences for the D, K, and L loci of the BALB/c mouse haplotype H-2^d^ was performed using prediction programs (Table 1). We selected and synthesized 12 peptides for subsequent evaluation considering the scores and frequency of peptides in each prediction programs (Table 2).

**Figure 4:**
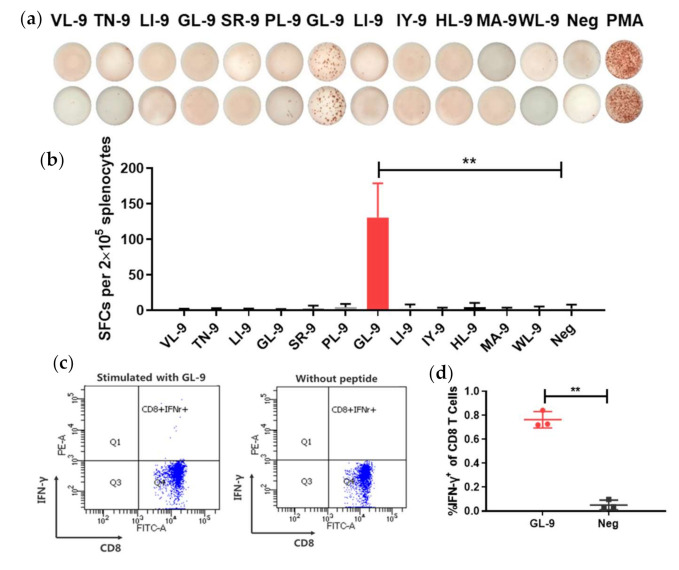
Evaluation of H-2^d^-restricted epitopes in LASV GPC. (**a**,**b**) BALB/c mice were immunized with Ad5-GPC_LASV_ at day 0. Splenocytes were extracted at 14 days post immunization and stimulated with the different peptides from LASV GPC for interferon (IFN)-γ enzyme-linked immunospot (ELISPOT) assays. (**c**,**d**) To further verify the identified peptide, splenocytes were stimulated with GL-9, and CD8^+^ T cells were assayed by intracellular IFN-γ staining. Abbreviations: IFN-γ, interferon γ; SFCs, spot forming cells. Data are presented as mean ± SD, and statically significant differences (Student’s *t*-test) are marked by bars and asterisks (** *p* < 0.01).

To identify specific class-I MHC H-2^d^ epitopes in LASV-GPC, a single injection of Ad5-GPC_LASV_ was administered to induce a cellular immune response. Splenocytes were extracted at 14 days post immunization and then stimulated for 24 h with different peptides in an interferon (IFN)-γ ELISPOT assay. We counted the number of spot forming cell (SFC) to indicate the stimulation ability of the peptide. Of the 12 selected peptides, only GL-9 (GYCLTRWML) showed specific induction of an IFN-γ response (Figure 4a,b).

To further verify whether GL-9 was a specific CD8^+^ epitope, BALB/c mice were immunized with Ad5-GPC_LASV_. At 14 days post immunization, splenocytes were stimulated for 6 h with GL-9 or without peptides, followed by intracellular IFN-γ staining and flow cytometry (Figure 4c,d). It was shown that GL-9 could activate CD8^+^ T cells from mice immunized with Ad5-GPC_LASV_, leading to a robust IFN-γ response. The cells without stimulation or cells from mice immunized with control had no detectable IFN-γ responses. Thus, GL-9 represents a H-2^d^-restricted CD8^+^ T-cell epitope in LASV GPC, and it was used as a candidate to test CD8^+^ T-cell responses to the LF vaccine in the BALB/c mouse model.

### 3.5. Analysis of the CD8^+^ T-Cell Responses

To identify LASV-specific CD8^+^ T-cell responses to Ad5-GPC_LASV_, BALB/c mice (*n* = 10 per group) were immunized intramuscularly with a dose of 10^7^ PFU of Ad5-GPC_LASV_ once on day 0, or twice, on days 0 and 28. The levels of anti-LASV GPC-specific IFN-γ, tumor necrosis factor (TNF)-α, IL-2, and CD107a were measured with multiparameter intracellular cytokine staining on days 14 and 42.

**Figure 5:**
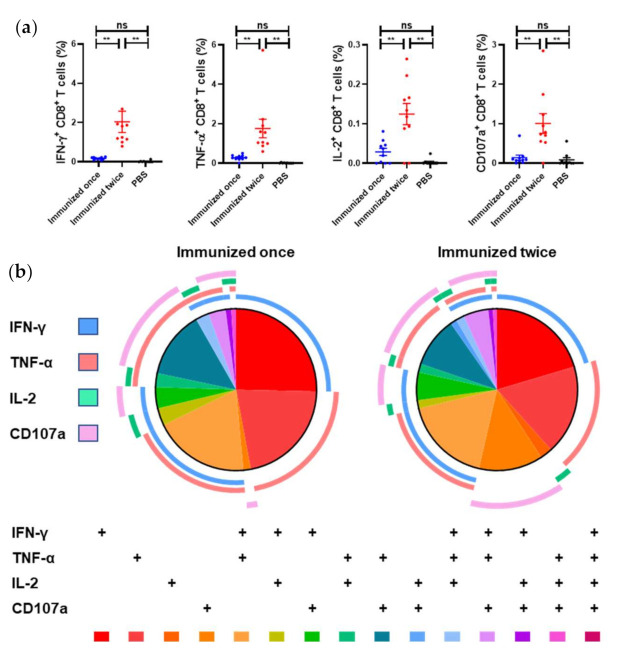
LASV GPC–specific CD8^+^ T-cell polyfunctionality response elicited by Ad5-GPC_LASV_. BALB/c mice (*n* = 10 per group) were immunized intramuscularly with a dose of 10^7^ PFU of Ad5-GPC_LASV_ once on day 0, or twice, on days 0 and 28. The murine splenocytes were collected on days 14 and day 42. (**a**) The CD8^+^ T cell-mediated immune responses were assessed by multiparameter intracellular cytokine staining. (**b**) SPICE plots were generated to demonstrate the fraction of LASV GPC-specific CD8^+^ T cells that were producing each immune marker in response to GL-9. Pie wedge colors refer to the legend below, whereas pie arc colors refer to the legend on the left. Abbreviations: IFN-γ, interferon γ; IL-2, interleukin 2; TNF-α, tumor necrosis factor α; CD107a, cluster of differentiation 107a. Data are presented as mean ± SEM, and statistical significance was determined by one way ANOVA and Turkey’s multiple comparison test (** *p* < 0.01).

At two weeks post vaccination, the mice developed weak anti-LASV GPC cellular immune responses (Figure 5a) as evidenced by the higher mean percentage of IFN-γ-, TNF-α-, and IL-2-positive CD8^+^ T cells in the vaccinated mice compared with the PBS-vaccinated control mice, but difference was not significant. After a boost immunization, CD8^+^ T-cells exhibited higher (*p* < 0.005) expression of IFN-γ, TNF-α, IL-2, and CD107a and became polyfunctional. Approximately 50% of the LASV GPC CD8^+^ T cells expressed >1 cytokine upon stimulation.

### 3.6. Generation of the LASV Pseudovirus and Detection of Neutralizing Antibodies (nAbs) in Mice Serum

**Figure 6:**
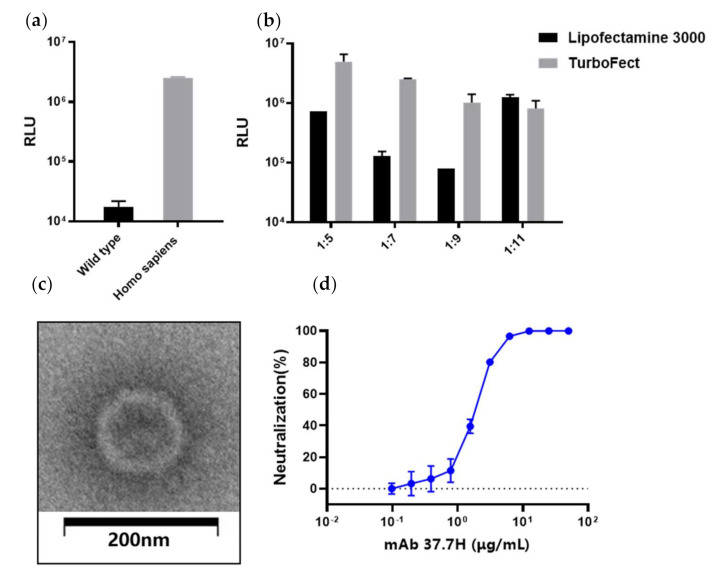
LASV pseudovirus construction and packaging condition optimization. Optimization of the LASV GPC gene (**a**); transfection reagents and the ratio of shuttle vector pDC316-LASV-GPC and human immunodeficiency virus (HIV) backbone plasmid pNL4-3.Luc-R−E− (**b**). (**c**) Electron micrographs of LASV pseudoviruses. The LASV pseudoviruses were imaged after staining with saturated uranyl acetate. Scale bar: 200 nm. (**d**) LASV pseudovirus neutralizing experiment using 37.7H.

LASV pseudovirus bearing the full-length GPC was constructed using the co-transfection 293T cells with LASV GPC expression plasmid and envelope-deleted HIV backbone plasmid. To improve the generation efficiency of LASV pseudovirus, we optimized several parameters, including the GPC codon usage, transfection reagents, and the mixture ratio of both plasmids (Figure 6a,b). Co-transfection of pDC316-LASV-GPC-opti and pNL4-3.Luc-R−E− at a ratio of 1:5 with TurboFect showed the highest relative light units (RLUs).

To evaluate pseudovirus assembly, the LASV pseudoviruses were investigated using negative staining electron microscopy. The results show that most of the particles were similar to HIV in morphology with a diameter of about 120 nm (Figure 6c). Moreover, 37.7H inhibited infection of LASV pseudovirus in a dose-dependent manner (Figure 4d). Using the pseudovirus-based neutralizing antibody method, we tested the neutralizing antibodies (nAbs) in the serum from the Ad5-GPC_LASV_ immunization group. All sera were negative for nAbs (data not shown).

### 3.7. Protective Effects of Vaccines against LASV Pseudovirus Infection in Mice

**Figure 7:**
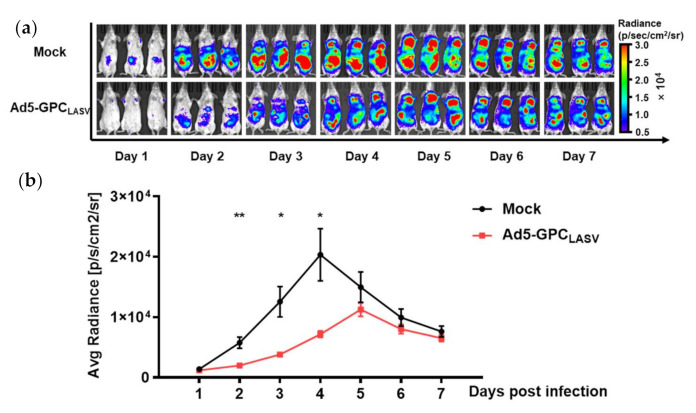
Ad5-GPC_LASV_ provided protection against LASV pseudovirus infection in mice. BALB/c mice (*n* = 3 per group) were intramuscularly (i.m.) inoculated with a single dose of 10^7^ PFU of Ad5-GPC_LASV_, followed by intraperitoneal (i.p.) inoculation with 10^5^ TCID_50_ LASV pseudovirus after 28 days. Bioluminescent images were acquired 1 day later (**a**). Values for average radiance at different time points are shown (**b**). Data are presented as mean ± SEM, and statistical significant differences (Student’s *t*-test) are marked by bars and asterisks (* *p* < 0.05; ** *p* < 0.01).

At 4 weeks after vaccination with a single dose of 10^7^ PFU of Ad5-GPC_LASV_, mice were challenged with an i.p. injection of 10^5^ TCID_50_ LASV pseudovirus, and the photo flux was detected one day later. The luciferase gene contained in LASV pseudovirus was first detected at two days post infection (dpi), peaking at 5 dpi in the immunized mice, whereas the reporter protein was first expressed at 1 dpi, peaking at 4 dpi in control mice. Although no nAbs were detected in vitro, the mice also had significantly lower bioluminescence intensity (threefold lower than control mice; *p* < 0.01), with the time of peak luminescence postponed (Figure 7a,b). Hence, vaccination of Ad5-GPC_LASV_ appeared to inhibit LASV pseudovirus infection.

## 4. Discussion

LF is an acute lethal viral disease associated with hemorrhagic manifestations. The lack of approved medical countermeasures highlights the urgent need to develop a safe and effective vaccine. For this purpose, we developed an LF vaccine on the basis of the clinically tested Ad5 vector. Our data show that Ad5-GPC_LASV_ is capable of inducing GPC-specific antibody responses. Moreover, modest GPC-specific CD8^+^ T-cell responses were detected and characterized mainly by the production of IFN-γ and TNF-α cytokines. Although no nAbs were detected in vitro, Ad5-GPC_LASV_ provided protection against LASV pseudovirus infection in mice.

So far, significant progress has been made in the development and preclinical research of LF vaccines. Recently, Junki Maruyama [32] et al. developed an LF vaccine utilizing the Ad5 vector platform with deletions in the E1, E2, and E3 genes expressing the NP or GPC. Animals immunized with both single-vectored Ad5 (E1-, E2b-) LASV GPC and NP vaccines produced antibodies after two doses. All vaccinated guinea pigs were protected from subsequent lethal LASV challenge. However, the T-cell-mediated response induced by Ad5 (E1-, E2b) was not characterized in that study. Furthermore, several findings have indicated that the T-cell response is correlated with protection and plays a dominant role in LASV control [33].

CD8^+^ T cells play a vital role in controlling LASV infections. They recognize viral MHC-I epitopes on the surface of infected cells, and either kill cells directly or via secretion of cytokines such as IFN-γ and TNF-α [15,34]. In this study, by using a hybrid immune-computational approach, we identified an H-2^d^-restricted CD8^+^ T-cell epitope GL-9 (GYCLTRWML) as a candidate to assay specific CD8^+^ T-cell responses of the LF vaccine based on GPC in the BALB/c mouse model. Through comparison with an overlapping peptide library, we offered a cost-effective method for further evaluation.

Ad5-GPC_LASV_ induced moderate levels of GPC-specific antibodies; nevertheless, it did not induce detectable levels of nAbs. The role of humoral immune responses during LASV infection is controversial. In one study, protection and recovery were not associated with nAbs, which appeared late and weak during infection [35]. Despite these issues, the significance of the humoral response in protection may be understated. The use of human monoclonal antibodies in both guinea pig and NHP models of LF was shown to be protective [17,18,27]. These monoclonal antibodies have a high neutralizing index, potentially suggesting that a strong neutralizing response may be protective. Therefore, vaccines capable of inducing a high titer of neutralizing antibodies may be feasible for protection against LASV. The recent explosion of atomic-level complex structures of LASV GPC with neutralizing antibodies have provided unprecedented opportunities for structure-based rational vaccine design [26,36]. LASV GPC is a class I viral fusion protein and is present in a labile, metastable form at the surface of infectious virions. Moreover, most neutralizing human monoclonal antibodies recognize prefusion GPC trimers. Taken together, engineering stabilized metastable prefusion GPC is paramount for the development of potentially protective vaccines for LASV [37,38,39,40].

On the other hand, the possibility that non-nAbs may also play a role in protection cannot be ignored. A recent study of an inactivated recombinant Lassa–rabies vaccine provided evidence for the role of non-nAbs in virus clearance via antibody-dependent cellular cytotoxicity (ADCC) and antibody-dependent cellular phagocytosis (ADCP) [41]. In our study, although no nAbs were detected, Ad5-GPC_LASV_ provided protection against LASV pseudovirus infection in mice. In this bioluminescence mouse model, the pseudovirus did not express GPC endogenously upon LASV pseudovirus infection. Antigens cannot be presented to CD8^+^ T cells through MHC class I pathways; thus, non-neutralizing antibodies may play a key role in inhibiting pseudovirus infection.

Overall, Ad5-GPC_LASV_ represents a potential vaccine candidate against Lassa fever, and further investigations will be needed to confirm its complete efficacy, safety, and immunogenicity in nonhuman primates (NHPs) when used against authentic LASV.

## Figures and Tables

**Table 1:** Programs used in this study for H-2^d^-restricted cluster of differentiation (CD8)^+^ T-cell epitope prediction.

Programs	URL
IEDB (SMM)	http://tools.immuneepitope.org/main/
NetCTL	https://services.healthtech.dtu.dk/service.php?NetCTL-1.2
NetMHC	https://services.healthtech.dtu.dk/service.php?NetMHC-4.0
PREDEP	http://margalit.huji.ac.il/Teppred/mhc-bind/index.html
ProPred-I	http://www.imtech.res.in/raghava/propred1/

**Table 2:** The predicted H-2^d^-restricted CD8^+^ T-cell epitopes in LASV GPC.

	Peptides	Amino Acid Sequence
GP1	59–67	TSLYKGVYE (TE-9)
	63–71	KGVYELQTL (KL-9)
	81–89	TMPLSCTKN (TN-9)
	93–101	HYIMVGNET (HT-9)
	237–245	SPIGYLGLL (SL-9)
	238–244	PSPIGYLGL (PL-9)
GP2	260–268	GTFTWTLSD (GD-9)
	277–285	GYCLTRWML (GL-9)
	293–301	FGNTAVAKC (FC-9)
	315–323	LFDFNKQAI (LI-9)
	361–369	IPYCNYSKY (IY-9)
	380–388	TSLPKCWLV (TT-9)
